# Association Analysis of Candidate Variants in Admixed Brazilian Patients With Genetic Generalized Epilepsies

**DOI:** 10.3389/fgene.2021.672304

**Published:** 2021-07-08

**Authors:** Felipe S. Kaibara, Tânia K. de Araujo, Patricia A. O. R. A. Araujo, Marina K. M. Alvim, Clarissa L. Yasuda, Fernando Cendes, Iscia Lopes-Cendes, Rodrigo Secolin

**Affiliations:** ^1^Department of Translational Medicine, School of Medical Sciences, University of Campinas (UNICAMP), Campinas, Brazil; ^2^Brazilian Institute of Neuroscience and Neurotechnology (BRAINN), Campinas, Brazil; ^3^Department of Neurology, School of Medical Sciences, University of Campinas (UNICAMP), Campinas, Brazil

**Keywords:** neurology, genetic generalized epilepsies, population genomics, admixed population, association studies

## Abstract

Genetic generalized epilepsies (GGEs) include well-established epilepsy syndromes with generalized onset seizures: childhood absence epilepsy, juvenile myoclonic epilepsy (JME), juvenile absence epilepsy (JAE), myoclonic absence epilepsy, epilepsy with eyelid myoclonia (Jeavons syndrome), generalized tonic–clonic seizures, and generalized tonic–clonic seizures alone. Genome-wide association studies (GWASs) and exome sequencing have identified 48 single-nucleotide polymorphisms (SNPs) associated with GGE. However, these studies were mainly based on non-admixed, European, and Asian populations. Thus, it remains unclear whether these results apply to patients of other origins. This study aims to evaluate whether these previous results could be replicated in a cohort of admixed Brazilian patients with GGE. We obtained SNP-array data from 87 patients with GGE, compared with 340 controls from the BIPMed public dataset. We could directly access genotypes of 17 candidate SNPs, available in the SNP array, and the remaining 31 SNPs were imputed using the BEAGLE v5.1 software. We performed an association test by logistic regression analysis, including the first five principal components as covariates. Furthermore, to expand the analysis of the candidate regions, we also interrogated 14,047 SNPs that flank the candidate SNPs (1 Mb). The statistical power was evaluated in terms of odds ratio and minor allele frequency (MAF) by the genpwr package. Differences in SNP frequencies between Brazilian and Europeans, sub-Saharan African, and Native Americans were evaluated by a two-proportion Z-test. We identified nine flanking SNPs, located on eight candidate regions, which presented association signals that passed the Bonferroni correction (rs12726617; rs9428842; rs1915992; rs1464634; rs6459526; rs2510087; rs9551042; rs9888879; and rs8133217; *p*-values <3.55e^–06^). In addition, the two-proportion Z-test indicates that the lack of association of the remaining candidate SNPs could be due to different genomic backgrounds observed in admixed Brazilians. This is the first time that candidate SNPs for GGE are analyzed in an admixed Brazilian population, and we could successfully replicate the association signals in eight candidate regions. In addition, our results provide new insights on how we can account for population structure to improve risk stratification estimation in admixed individuals.

## Introduction

Genetic generalized epilepsies (GGEs) are a group of epilepsy syndromes in which the main feature is the recurrence of generalized onset seizures with no known or suspected etiology other than possible genetic predisposition (Berg et al., 2010; Scheffer et al., 2017). GGEs are among the most common types of epilepsy, with an estimated prevalence of 190 per 100,000 individuals (Aaberg et al., 2017). They include well-established syndromes: childhood absence epilepsy, juvenile myoclonic epilepsy (JME), juvenile absence epilepsy (JAE), myoclonic absence epilepsy (a rare form of GGE), epilepsy with eyelid myoclonia (Jeavons syndrome), generalized tonic–clonic seizures, and generalized tonic–clonic seizures alone (Berg et al., 2010). These different GGE syndromes share most genetic susceptibility factors, suggesting an important correlation among the clinical subtypes (International League Against Epilepsy Consortium on Complex Epilepsies (ILAE Consortium on Complex Epilepsies)., 2018). The diagnosis of GGE relies mainly on clinical information and electroencephalographic examination (Scheffer et al., 2017).

Previous genome-wide association studies (GWASs) and exome sequencing analyses have identified 48 single-nucleotide polymorphisms (SNPs) putatively associated with susceptibility to the GGEs (EPICURE Consortium et al., 2012a,b; International League Against Epilepsy Consortium on Complex Epilepsies (ILAE Consortium on Complex Epilepsies), 2014; International League Against Epilepsy Consortium on Complex Epilepsies (ILAE Consortium on Complex Epilepsies)., 2018; Zhang et al., 2014; Wang et al., 2019). These SNPs are located in or near several genes encoding ion channels and synaptic vesicles, making them plausible candidates for the susceptibility to epilepsy (International League Against Epilepsy Consortium on Complex Epilepsies (ILAE Consortium on Complex Epilepsies)., 2018). However, most of these studies evaluated non-admixed populations, including five studies based on Europeans, three based on Asian populations, mainly Chinese, and two based on African populations (EPICURE Consortium et al., 2012a,b; International League Against Epilepsy Consortium on Complex Epilepsies (ILAE Consortium on Complex Epilepsies), 2014; International League Against Epilepsy Consortium on Complex Epilepsies (ILAE Consortium on Complex Epilepsies)., 2018; Zhang et al., 2014; Wang et al., 2019). It is well known that admixed American populations are underrepresented in GWASs, decreasing the accuracy of replicating, predicting, and estimating polygenic risks for complex disorders in these populations (Martin et al., 2017, 2019).

Therefore, this work aims to investigate if a genetic association exists between previously reported candidate SNPs and GGEs in a cohort of admixed Brazilians. To accomplish this goal, we first investigated the population structure of Brazilian patients with GGE. Subsequently, we performed an association study using the 48 previously reported candidate SNPs and their flanking regions.

## Materials and Methods

### Subjects

We evaluated a total of 87 patients with GGE who were followed up prospectively in the outpatient epilepsy clinic of the University of Campinas (UNICAMP) hospital. All patients had the diagnosis of GGE according to criteria established by the International League Against Epilepsy (ILAE) (Berg et al., 2010; Fisher et al., 2014). Patients were compared with a group of 340 individuals without any neurological disorder from the BIPMed database (Rocha et al., 2020). Both samples are predominantly from the Southeastern region in Brazil. Among the patients with GGE, we found 63 with JME, 10 with JAE, four generalized tonic–clonic seizures alone, two with Jeavons syndrome, one with myoclonic absence epilepsy, one with epilepsy with generalized tonic–clonic seizures, and six patients in whom a specific GGE syndrome could not be determined. All research participants signed an informed consent form previously approved by our Institutional Research Ethics Committee (IRB # 12112913.3.0000.5404).

### Single-Nucleotide Polymorphism Quality Control and Population Structure Analysis

We extracted the genotypes for the 48 candidate SNPs (Table 1) from the SNP-array data generated by the Genome-Wide Human SNP Array 6.0 (Affymetrix Inc., Thermo Fisher Scientific, Waltham, MA, United States). These SNP-array data contain 905,171 available SNPs (GRCh37 build). To obtain an unbiased estimation of the population structure of our samples, we processed the SNP-array dataset of the 87 patients with GGE and the 340 BIPMed controls according to previous processing recommendations and pipelines (Anderson et al., 2010; Secolin et al., 2019). First, we removed ambiguous variants (with G/C or A/T alleles) from each dataset. Next, we merged the two datasets into one larger admixed Brazilian dataset (*N* = 427), maintaining only biallelic SNPs, autosomal SNPs, SNPs without Hardy–Weinberg disequilibrium (*p*-value <0.000001), and missing data <10%. Then, we estimated the heterozygosity rate for each sample and removed individuals with heterozygosity rates higher or lower than three standard deviations from the mean to avoid individuals with high inbreeding (low heterozygosity rates) or sample contamination (high heterozygosity rates). We also removed pairs of individuals who presented a proportion of identical-by-state (IBS) alleles >0.85, which could indicate duplicated samples, and individuals with genomic relatedness matrix estimations higher than 0.125, which is the expected genomic relatedness for third-degree relatives (Anderson et al., 2010). The merging process, genotyping, and sample filtering were performed using PLINK 1.9 software (Purcell et al., 2007).

**TABLE 1:** Descriptive statistics and logistic regression analysis of the candidate SNPs and the nine flanking SNPs that passed the most stringent Bonferroni correction (*p*-value = 3.55e^–06^; in bold).

Chr	Position (BP)	dbSNP	A1/A2	Reference (PMID)	Reference effect sizes	MAF (A1)	HWE *p*-value	OR (95% CI) (A1)	Nominal *p-*value
1	10046460	rs12136213*	G/A	22242659	**–**	0.268	0.7882	0.76 (0.49–1.19)	0.2285
1	239970097	rs12059546	G/A	25271899; 22949513	1.42 (1.26–1.61)	0.236	0.0562	0.86 (0.56–1.32)	0.4876
**1**	**240605694**	**rs12726617**	**C/T**	**–**	**–**	**0.412**	**0.0015**	**2.44 (1.96–3.54)**	**2.62e^–06^**
**1**	**240610720**	**rs9428842**	**A/G**	**–**	**–**	**0.052**	**0.6117**	**5.86 (2.81–12.2)**	**2.29e^–06^**
2	23898317	rs4665630	C/T	22242659	**–**	0.176	0.4723	1.11 (0.68–1.82)	0.6693
2	57934055	rs13026414*	T/C	25271899; 22242659	1.51 (0.81–2.83)	0.349	1.0000	0.97 (0.64–1.46)	0.8705
2	57950346	rs4671319*	G/A	22242659	**–**	0.422	0.1598	0.70 (0.47–1.04)	0.0765
2	58042241	rs1402398*	G/A	22242659	**–**	0.35	0.0147	0.77 (0.52–1.14)	0.1906
2	58051769	rs12185644	C/A	22242659	**–**	0.333	0.1557	1.03 (0.70–1.53)	0.8749
2	58059803	rs2947349*	C/A	25087078	1.23 (1.16–1.31)	0.307	0.0064	0.83 (0.55–1.25)	0.3731
2	145359909	rs10496964	T/C	25271899; 22949513	0.68 (0.60–0.78)	0.138	0.8271	1.29 (0.77–2.17)	0.3388
2	145381225	rs13020210*	G/A	22242659	**–**	0.213	0.4299	1.15 (0.73–1.80)	0.5395
2	166943277	rs11890028*	G/T	25271899; 22949513; 22242659	0.85 (0.79–0.92)	0.257	0.3361	1.08 (0.69–1.69)	0.7323
2	167084615	rs13406236*	C/T	22242659	**–**	0.294	1.0000	0.96 (0.63–1.46)	0.8425
2	191583507	rs887696*	C/T	22242659	**–**	0.401	0.8268	1.28 (0.86–1.88)	0.2198
**3**	**61699969**	**rs1915992**	**A/G**	**–**	**–**	**0.221**	**0.6469**	**3.62 (2.27–5.76)**	**5.77e^–08^**
3	61733962	rs624755	G/T	22242659	**–**	0.368	1.0000	0.81 (0.53–1.22)	0.3040
3	63075267	rs1374679	C/T	22242659	**–**	0.379	0.315	0.97 (0.65–1.46)	0.8945
3	66326302	rs782728*	A/G	22242659	**–**	0.468	1.0000	0.89 (0.61–1.30)	0.5499
**3**	**167113205**	**rs1464634**	**T/G**	**–**	**–**	**0.186**	**0.0026**	**4.03 (2.35–6.93)**	**4.48e^–07^**
3	167861408	rs111577701*	T/C	25087078	1.16 (1.09–1.24)	0.074	0.4246	0.90 (0.43–1.89)	0.7834
4	31147874	rs1044352	T/G	25087078; 22242659	1.13 (1.12–1.23)	0.454	0.3963	1.17 (0.79–1.73)	0.4305
4	31151357	rs28498976*	A/G	22242659	**–**	0.446	0.2002	1.26 (0.84–1.88)	0.2629
4	46240287	rs535066*	G/T	22242659	**–**	0.413	1.0000	0.95 (0.64–1.41)	0.8021
4	46397617	rs11943905*	T/C	22242659	**–**	0.293	0.3092	1.19 (0.80–1.78)	0.3828
5	114221505	rs4596374*	C/T	22242659	**–**	0.475	0.0263	0.89 (0.62–1.29)	0.5498
5	114268470	rs55670112*	C/A	25087078	1.18 (1.10–1.26)	0.482	0.0458	1.19 (0.83–1.72)	0.3456
5	150840380	rs357608*	T/C	22242659	**–**	0.481	0.2452	0.97 (0.67–1.40)	0.8506
5	162867195	rs2069347	C/T	22242659	**–**	0.475	0.8331	1.18 (0.80–1.73)	0.3981
5	166893257	rs1025482*	C/T	22242659	**–**	0.488	0.4619	1.03 (0.71–1.49)	0.8813
5	166932520	rs1432881	T/C	22242659	**–**	0.407	0.2745	1.03 (0.69–1.54)	0.8899
5	167913510	rs244903*	G/A	22242659	**–**	0.364	0.4278	1.14 (0.76–1.71)	0.5419
6	16971575	rs68082256*	A/G	22242659	**–**	0.183	0.2934	0.76 (0.44–1.29)	0.3077
**6**	**17155461**	**rs6459526**	**T/C**	**–**	**–**	**0.222**	**0.0934**	**3.57 (2.15–5.92)**	**8.49e^–07^**
6	128309768	rs13200150*	G/A	22242659	**–**	0.222	0.1711	0.92 (0.57–1.49)	0.7490
11	102595135	rs1939012*	A/G	25087078; 22242659	1.12 (1.07–1.17)	0.446	0.6695	0.60 (0.40–0.90)	0.0133
**11**	**102948592**	**rs2510087**	**A/G**	**–**	**–**	**0.217**	**0.0865**	**3.17 (1.96–5.14)**	**2.76e^–06^**
13	23966145	rs1008812*	A/G	22242659	**–**	0.465	1.0000	0.86 (0.59–1.26)	0.4385
**13**	**24615989**	**rs9551042**	**A/G**	**–**	**–**	**0.243**	**0.1530**	**0.22 (0.11–0.41)**	**2.81e^–06^**
13	91417190	rs1332470	C/T	22242659	**–**	0.307	0.0001	1.10 (0.76–1.58)	0.6244
16	30914626	rs1046276	T/C	22242659	**–**	0.331	1.27e^–13^	–	-
**16**	**31310372**	**rs9888879**	**C/T**	**–**	**–**	**0.317**	**0.3935**	**3.65 (2.27–5.85)**	**7.91e^–08^**
16	50045839	rs4638568	A/G	22242659	**–**	0.079	0.7125	1.64 (0.84–3.18)	0.1466
17	46027565	rs12951323*	A/C	22949513; 22242659	0.79 (0.72–0.86)	0.206	0.1075	0.67 (0.39–1.15)	0.1429
17	46045495	rs4794333*	A/G	22242659	**–**	0.356	0.0511	0.64 (0.42–0.97)	0.0361
17	46123004	rs72823592*	A/G	25271899; 22949513	0.77 (0.71–0.83)	0.181	0.7209	0.65 (0.37–1.13)	0.1262
18	48402338	rs2665558*	T/C	30719716; 22242659	**–**	0.451	0.6707	0.95 (0.64–1.40)	0.7980
18	48404784	rs2255610*	G/A	30719716; 22242659	**–**	0.474	0.7518	1.14 (0.77–1.67)	0.5199
18	48407326	rs608781*	C/T	30719716; 22242659	**–**	0.118	0.4472	1.72 (0.98–3.02)	0.0610
18	48414235	rs2850545*	A/C	30719716; 22242659	**–**	0.471	0.5976	1.12 (0.75–1.65)	0.5845
18	48456903	rs645088	T/C	30719716; 22242659	**–**	0.339	0.4812	0.83 (0.55–1.26)	0.3873
18	48458662	rs649224	A/G	30719716; 22242659	**–**	0.107	0.7821	1.50 (0.83–2.70)	0.1834
18	48464204	rs654136	T/C	30719716; 22242659	**–**	0.488	0.5266	1.02 (0.69–1.51)	0.9271
19	53719250	rs9788	A/G	22242659	**–**	0.315	0.9032	1.57 (1.05–2.34)	0.0274
21	32183996	rs2833098*	G/A	22242659	**–**	0.369	0.9099	1.11 (0.75–1.64)	0.6152
**21**	**48063151**	**rs8133217**	**G/A**	**–**	**–**	**0.214**	**0.0422**	**0.15 (0.07–0.32)**	**4.94e^–07^**
21	48077812	rs2839377	T/C	22242659	**–**	0.497	0.4605	1.07 (0.72–1.57)	0.7491

The positions are based on GRCh37. SNPs with an asterisk (*) were obtained by BEAGLE imputation.

*BP, base pairs; PMID, PUBMED ID publications; MAF, minor allele frequency; HWE, Hardy–Weinberg equilibrium; OR, odds ratio; CI, confidence interval.*

Subsequently, we merged the filtered admixed Brazilian sample with the 1000 Genomes Project (1KGP) dataset (The 1000 Genomes Project Consortium et al., 2015), maintaining the SNPs present only in the admixed Brazilian sample. After merging, we removed SNPs with a minor allele frequency (MAF) < 0.01 and SNPs in linkage disequilibrium (LD), using the following parameters: window size = 50 SNPs, shift step = 5 SNPs, and *r*^2^ = 0.5 (Anderson et al., 2010). We compared our dataset with the 1KGP data by principal component analysis (PCA) using PLINK v1.9 software (Purcell et al., 2007) to evaluate the presence of population-based outliers in the Brazilian samples.

To evaluate whether patients with GGE and BIPMed controls present population stratification, we performed the analysis of molecular variance (AMOVA) (Excoffier et al., 1992) using the poppr.amova R package and the RStudio interface, comparing the genetic distance among the two groups based on a set of 10,000 random SNPs across the genome. The AMOVA partitions the source of genetic variance (σ^2^) into two components: within-groups and between-groups. The null hypothesis states that the samples were obtained from a global population, with variation due to random sampling in the construction of populations. Thus, we would expect a high heterogeneity within groups (σ^2^ = 100%) and no heterogeneity between groups (σ^2^ = 0%). On the other hand, under the alternative hypothesis, each group was obtained from different populations, and we would expect a low heterogeneity within groups (σ^2^ < 100%) and high heterogeneity between groups (σ^2^ > 0%) (Excoffier et al., 1992). Therefore, to evaluate the significance of σ^2^ components, we generated a Monte Carlo null distribution of 10,000 variance components and tested against the observed variance components by the randtest function in the ade4 R package.

### Single-Nucleotide Polymorphism Selection and Imputation

We observed that 31 SNPs were not found in the SNP-array dataset. Therefore, we performed an imputation of all 48 SNPs to obtain the missing SNPs and to evaluate the concordance between the imputed genotypes and the genotypes assessed by the SNP array. Since we analyzed a sample of admixed individuals, we elected to perform the imputation using two approaches. First, we phased and imputed the dataset using SHAPEIT2 v2.r387 (O’Connell et al., 2014) and BEAGLE v5.1 software (Browning et al., 2018) using the default software parameters for phasing and imputation. As a reference for the BEAGLE imputation, we used the 1KGP dataset (GRCh37/hg19 assembly) (The 1000 Genomes Project Consortium et al., 2015). To save on computation time, we imputed only the chromosomes in which the candidate SNPs are located (Table 1). We also evaluated whether the genotypes were successfully imputed by the correlation (in terms of *r*^2^) of genotype dosage values between the imputed genotypes and true genotypes used as a reference from the 1KGP provided by the BEAGLE software. For the second imputation approach, we used the TOPMED Imputation Server (Das et al., 2016), with the TOPMed v.R2 on GRCh38 build (Kowalski et al., 2019). The TOPMED server imputation performed the liftover from GRCh37 to GRCh38 and the phasing using the EAGLE v.2.4 algorithm. Finally, imputation was performed by minimac4.

### Candidate Single-Nucleotide Polymorphism Association Analysis

After genotype and individual filtering, 360 individuals remained (69 patients with GGE and 291 BIPMed controls), which were used in the association analysis. We estimated the statistical power of our sample by the genpwr package in R (Moore et al., 2020), which analyzes the statistical power under the evaluation between true and test genetic models (Dominant, Additive, Recessive, 2df/unspecified model). In this case, we evaluate the statistical power using a vector of MAFs (from 0.05 to 0.45, by 0.05) and an odds ratios (from 1.5 to 2.0, by 0.1) since not all candidate SNPs presented OR estimations from previous studies. We also set the following parameters for genpwr: model = logistic; N = 360; case/control ratio = 69/291 = 0.237; and alpha = 0.05.

We evaluated candidate SNP association and OR estimation by logistic regression analysis using the PLINK v1.9 software (Purcell et al., 2007), including the first five PCs as covariates. We did not include age, age at seizure onset, and sex since these variables have not been correlated with the GGE phenotype (Berg et al., 2010; Scheffer et al., 2018).

It has been reported that SNPs found to be associated with the phenotype by GWAS in one population may be only nominally associated or non-associated in another population due to difference in LD across populations (Akiyama et al., 2019; Chen et al., 2020; Graff et al., 2021); however, it does not mean that an associated signal in the genomic region cannot be replicated. This is because the SNPs ascertained from GWAS are only tagging variants linked to causal ones. The lack of signals in the replication population could simply be caused by the broken linkage between tagging and causal variants. Therefore, to account for the difference in LD across populations and to investigate the transferability of previous GWAS signals, we used the SNP-array dataset, filtered for population structure and without LD pruning (652,883 SNPs), to interrogate the SNPs flanking the 1 Mb upstream and downstream the candidate SNPs by logistic regression. We assumed a *p-*value adjusted by the Bonferroni correction to avoid biased results due to the multiple comparisons. In this case, we used two thresholds: the first threshold took into account the 48 SNPs (*p-*value = 0.05/48 = 0.001), assuming one effective test per region, which is a reasonable assumption and may lead to more informative results. However, this threshold may not be stringent enough. Therefore, we also evaluate the results under a second threshold, considering all the 48 candidate SNPs and the additional flanking SNPs tested, and the results were plotted using the qqman package in R software (Turner, 2014).

Since previous studies of GGE were based on European populations and admixed Brazilians have a large proportion of European ancestry, we decided to evaluate whether the candidate SNP allele frequencies are similar between Brazilian and European populations. We extracted European allele frequencies from the gnomAD database (Karczewski et al., 2020) and performed a two-proportion Z-test using the prop.test function in R. Also, we included African populations from gnomAD in the analysis due to the sub-Saharan African ancestry component present in Brazilian populations. However, since gnomAD does not separate Native American populations in the database, we include the Latin population in the analysis as a proxy.

## Results

### Population Structure Analysis

**FIGURE 1:**
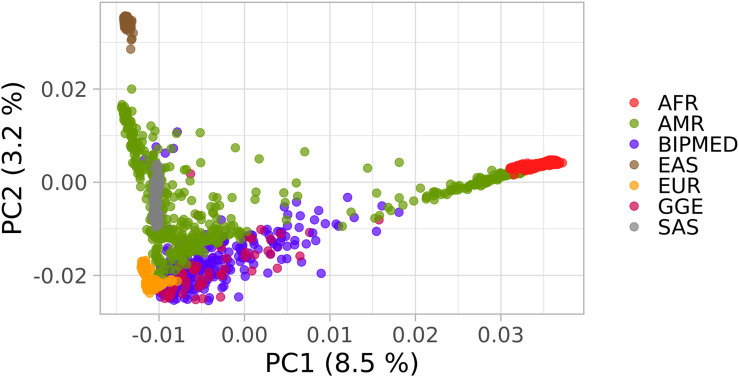
Principal component analysis (PCA) plot of the Brazilian samples and 1000 Genomes Project (1KGP) dataset. The *x*-axis and *y*-axis show the first and second principal components (PC1 and PC2) and their respective percentage variability. Each point represents one individual and each color indicates patients with genetic generalized epilepsy (GGE), BIPMed controls, and the continental populations described in 1KGP. as follows: Sub-Saharan Africans (AFR); Europeans (EUR); admixed Americans (AMR); Southwestern Asians (SAS); Eastern Asians (EAS).

The principal components in the PCA plot indicate that both cases and controls clustered together and were spread between Europeans, sub-Saharan Africans, and other admixed American populations (Figure 1). The AMOVA results showed that 99.61% of the genetic variation was observed within groups (patients or controls), and only 0.39% of the genetic variation was observed between groups (Table 2). Because we have one hierarchical level of stratification (patients/controls), the poppr.amova package provided one total φ-statistics = 0.0031, with a *p*-value = 0.001 (Table 2), indicating evidence of population stratification between patients and controls and the necessity of population structure correction in further association tests.

**TABLE 2:** AMOVA results.

Variance component	Variance σ^2^	Percentage of variance	Total Φ-statistics	*p*-Value
Between samples	0.1884	0.39	0.00393	0.001
Within samples	47.7333	99.61		
Total	47.9217	100.00		

*Variance component estimations are based on the genetic distance among patients with GGE and controls, including the Monte Carlo test *p*-value. Values were estimated based on 10,000 (10k) random autosome SNPs across the genome.*

*AMOVA, analysis of molecular variance; SNPs, single-nucleotide polymorphisms; GGE, genetic generalized epilepsy.*

### Single-Nucleotide Polymorphism Selection and Imputation

According to the imputation results from the BEAGLE software (Browning et al., 2018), the correlation between the estimated allele dosage and the true allele dosage from the 1KGP is used as reference (in terms of *r*^2^) and presented a minimum value of 95%. In addition, all 17 SNPs genotyped by the SNP array were correctly imputed by the BEAGLE software. However, we observed that the 17 SNPs genotyped by the SNP array presented only 45.7% of matching (on average) with genotypes imputed by the TOPMED server. Thus, we decided to perform further analysis using the imputed genotypes generated by the BEAGLE software.

### Candidate Single-Nucleotide Polymorphism Association Analysis

**FIGURE 2:**
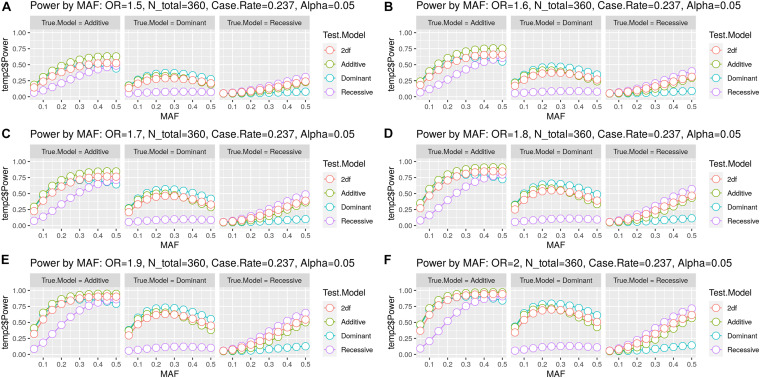
Statistical power estimation. The figure includes six panels **(A–F)** for the six OR values evaluated. Each panel shows the statistical power (*y*-axis) estimated by minor allele frequency (MAF) (*x*-axis) for the combination of the three true genetic models (Dominant, Additive, and Recessive) with the four test models (Dominant, Additive, Recessive, and 2df/unspecified genetic model). Each colored line represents the test model, and each point represents the MAF.

As detailed in Table 1, one candidate SNP (rs1046276) was withdrawn from further association analysis due to the presence of the Hardy–Weinberg disequilibrium (*p* < 0.000001). According to the analysis performed using the genpwr package (Moore et al., 2020), we observed that the Additive model presented the highest power estimation. We did not observe 80% of statistical power for OR ≤ 1.6 (≥ 0.62 for protection effect) (Figures 2A,B). However, we calculated that our study had 80% power to detect an increased risk in terms of OR ≥ 1.7 (≤0.58 for protection effect) with MAF > 0.25 (Figure 2C), OR ≥ 1.8 (≤0.55 for protection effect) with MAF > 0.2 (Figure 2D), and OR ≥ 1.9 (≤0.52 for protection effect) with MAF > 0.15 (Figures 2E,F).

**FIGURE 3:**
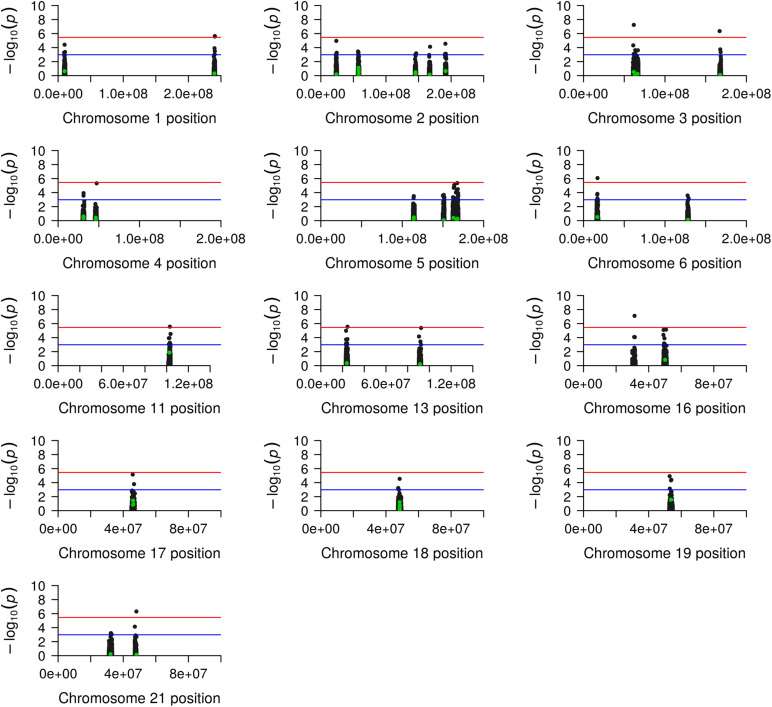
Manhattan plot of genetic generalized epilepsy (GGE) candidate regions. The figure shows a plot for each candidate chromosome, including the chromosome position (*x*-axis) and the –log10(*p-*value) in the *y*-axis. The green and black points represent the candidate and the flanking single-nucleotide polymorphisms (SNPs), respectively. The blue line indicates the suggestive association signal based on the *p*-value adjusted by the Bonferroni correction under the 48 candidate SNPs. The red line indicates the association signal based on the *p-*value adjusted by Bonferroni under the 48 candidate SNPs plus the 14,047 flanking SNPs.

We identified suggestive evidence of a protective effect for the SNP rs1939012^∗^A allele (MAF = 0.446; OR = 0.60; 95% CI = 0.40–0.90; nominal *p*-value = 0.0133) and rs4794333^∗^A allele (MAF = 0.356; OR = 0.64; 95% CI = 0.42–0.97; nominal *p*-value = 0.0361) and an increased risk for rs9788^∗^G (MAF = 0.315; OR = 1.57; 95% CI = 1.05–2.34, nominal *p*-value = 0.0274). However, these results did not pass the corrections for multiple comparisons by Bonferroni (Table 1). Interesting, we found 14,047 flanking SNPs, encompassing 29 candidate regions (Supplementary Data). As shown in Figure 3, under the *p-*value threshold = 0.001, we observed that the association signals in all candidate regions passed the Bonferroni correction. Adjusting the *p*-values by Bonferroni for 14,095 tests (*p*-value = 3.55e^–06^), we observed that nine flanking SNPs passed the multiple comparison adjustment: rs12726617 and rs9428842 on chromosome (chr.) 1q43; rs1915992 on chr. 3p14.2; rs1464634 on chr. 3q26.1; rs6459526 on chr. 6p22.3; rs2510087 on chr. 11q22.3; rs9551042 on chr. 13q12.12; rs9888879 on chr. 16p11.2; and rs8133217 on chr. 21q22.3 (Figure 3 and Table 1).

Since most Brazilian ancestry is derived from European populations (Kehdy et al., 2015; Moura et al., 2015; Secolin et al., 2019), we could hypothesize that effect sizes in terms of OR would present a higher correlation with European effect sizes comparing with Chinese or European/African American samples from previous studies (EPICURE Consortium et al., 2012a,b; International League Against Epilepsy Consortium on Complex Epilepsies (ILAE Consortium on Complex Epilepsies), 2014; International League Against Epilepsy Consortium on Complex Epilepsies (ILAE Consortium on Complex Epilepsies)., 2018; Zhang et al., 2014; Wang et al., 2019). Thus, we show a comparison of the OR estimations of 11 SNPs, which were available from the previous studies, and the OR estimations in our admixed Brazilian samples (Table 3). Remarkably, Chinese and European/African American samples also presented similar OR estimations compared with admixed Brazilians. Two SNPs had different OR estimations for admixed Brazilians compared with European and Chinese samples (rs10496964 and rs11890028).

**TABLE 3:** Odds ratio comparison among studies.

SNP	Brazil	Population from previous studies		
		
		European (PMID: 22949513)	Chinese (PMID: 25271899)	European/African Americans (PMID: 25087078)
rs12059546	0.86 (0.56–1.32)	1.53 (1.32–1.79)	0.93 (0.57–1.53)	–
rs13026414	0.97 (0.64–1.46)	0.78 (0.71–0.86)	1.51 (0.81–2.83)	–
rs2947349	0.83 (0.55–1.25)	–	–	1.23 (1.16–1.31)
rs10496964	1.29 (0.77–2.17)	0.63 (0.52–0.76)	0.50 (0.18–1.40)	–
rs11890028	1.08 (0.69–1.69)	0.77 (0.70–0.85)	0.77 (0.26–2.24)	–
rs111577701	0.90 (0.43–1.89)	–	–	1.16 (1.09–1.24)
rs1044352	1.17 (0.79–1.73)	–	–	0⋅88 (0⋅82–0⋅93)
rs55670112	1.19 (0.83–1.72)	–	–	1.18 (1.10–1.26)
rs1939012	0.60 (0.40–0.90)	–	–	1.12 (1.07–1.17)
rs12951323	0.67 (0.39–1.15)	0.75 (0.66–0.84)	–	–
rs72823592	0.65 (0.37–1.13)	0.74 (0.66–0.83)	–	–

*The table shows the OR estimation available in each study, including the 95% confidence interval in parentheses.*

*SNP, single-nucleotide polymorphism.*

Furthermore, the two-proportion Z-test results showed that 25 candidate SNPs have allele frequencies that were different when comparing admixed Brazilian and the ancestral populations. Among them, 16 SNPs presented differences in allele frequencies comparing admixed Brazilian and European populations. All 25 SNPs presented different allele frequencies when comparing admixed Brazilian and African samples. Remarkably, we also found 15 candidate SNPs with different allele frequencies when comparing admixed Brazilians and the Latin American samples in the gnomAD database (Table 4).

**TABLE 4:** Two-proportion Z-test results comparing Brazilian samples with European, African, and Latin-American samples from gnomAD.

SNP ID	Allele	Brazilian samples	European vs. Brazilian samples		African vs. Brazilian samples		Latin American vs. Brazilian samples	
					
		Allele frequency	Allele frequency	*p*-Value	Allele frequency	*p*-Value	Allele frequency	*p*-Value
rs12136213	G	0.268	0.282	1	0.105	3.66e^–37^	0.257	1
rs4665630	G	0.176	0.896	0	0.511	6.45e^–65^	0.895	6.43e^–178^
rs4671319	T	0.422	0.521	6.37e^–06^	0.096	2.79e^–55^	0.417	1
rs1402398	G	0.350	0.615	4.35e^–44^	0.708	3.69e^–114^	0.558	7.64e^–15^
rs2947349	G	0.307	0.617	5.11e^–60^	0.749	0	0.567	8.40e^–23^
rs10496964	C	0.138	0.163	1	0.590	3.37e^–35^	0.091	1.24e^–01^
rs13020210	C	0.213	0.832	0	0.922	1.36e^–41^	0.861	1.31e^–144^
rs11890028	A	0.257	0.278	1	0.036	3.32e^–05^	0.205	4.62e^–01^
rs887696	G	0.401	0.660	2.88e^–44^	0.479	1.36e^–83^	0.481	4.83e^–02^
rs111577701	G	0.074	0.131	2.21e^–04^	0.183	5.95e^–17^	0.057	1
rs28498976	C	0.446	0.379	8.41e^–03^	0.689	0.00019	0.313	2.12e^–06^
rs11943905	C	0.293	0.265	1	0.743	6.70e^–13^	0.184	1.25e^–05^
rs55670112	C	0.482	0.457	1	0.386	6.63e^–11^	0.541	5.62e^–01^
rs68082256	C	0.183	0.207	1	0.792	9.55e^–25^	0.209	1
rs1939012	A	0.446	0.498	1.82e^–01^	0.755	1.01e^–47^	0.517	1.60e^–01^
rs1008812	T	0.465	0.490	1	0.210	0.00884	0.579	2.24e^–04^
rs4638568	T	0.079	0.057	3.87e^–01^	0.553	7.79e^–20^	0.031	8.21e^–04^
rs12951323	A	0.206	0.788	9.49e^–280^	0.533	1.65e^–88^	0.882	2.92e^–158^
rs4794333	G	0.356	0.390	1	0.704	0.00814	0.290	1.62e^–01^
rs72823592	T	0.181	0.241	6.02e^–03^	0.177	6.01e^–09^	0.121	2.91e^–02^
rs608781	C	0.118	0.928	0	0.618	4.02e^–194^	0.900	2.81e^–208^
rs645088	C	0.339	0.625	5.61e^–52^	0.351	1.73e^–152^	0.781	4.82e^–68^
rs649224	T	0.107	0.074	2.08e^–02^	0.296	2.96e^–28^	0.064	1.13e^–04^
rs9788	G	0.489	0.617	4.18e^–14^	0.316	6.18e^–228^	0.609	9.30e^–06^
rs2839377	C	0.497	0.535	1	0.512	1.21e^–11^	0.463	1

**p*-Values are adjusted by the Bonferroni correction. We show the results for the 25 SNPs presenting significant differences in allele frequencies among populations. SNP, single-nucleotide polymorphism.*

## Discussion

The Brazilian population was formed by an admixture of three main ancestry populations: Europeans, sub-Saharan Africans, and Native Americans (Kehdy et al., 2015; Moura et al., 2015; Secolin et al., 2019). In this scenario, it is important to explore whether candidate SNPs previously identified as associated with complex disorders in non-admixed populations also display association signals in the Brazilian admixed population. By doing so, one can better estimate the impact of population structure in estimating polygenic risks, avoiding misinterpretation of risk scores calculated in other populations.

Previous genetic association studies have identified 48 candidate SNPs associated with GGEs (EPICURE Consortium et al., 2012a,b; International League Against Epilepsy Consortium on Complex Epilepsies (ILAE Consortium on Complex Epilepsies), 2014; International League Against Epilepsy Consortium on Complex Epilepsies (ILAE Consortium on Complex Epilepsies)., 2018; Zhang et al., 2014; Wang et al., 2019). These studies were all performed in non-admixed populations, predominantly of European ancestry, raising the question of reproducibility of these results in other populations. Lack of transferability of GWAS results and polygenic risk scores obtained from Europeans and American admixed populations have previously been reported (Martin et al., 2017, 2019), making it important to investigate whether these SNPs are associated with GGEs in our admixed Brazilian sample.

An alternative explanation for the lack of reproducibility among populations relies on the observation that only tagging SNPs are ascertained in GWAS, and the lack of replication in different populations could be due to broken linkage between the tagging SNPs and the causal variants (Akiyama et al., 2019; Chen et al., 2020; Graff et al., 2021). Thus, we searched for SNPs flanking 1 Mb upstream and downstream of the candidate regions to investigate this issue. Indeed, we found 14,047 flanking SNPs, and nine of them presented statistically significant association signals after stringent corrections for multiple comparisons (*p*-value < 3.55e^–06^). These nine SNPs encompass eight candidate regions (Table 2 and Figure 3), which were previously found associated in European samples (1q43; 3p14.2; 3q26.1; 6p22.3; 11q22.3; 13q12.12; 16p.11.2; and 21q22.3), and two of them were also found associated in a mixed sample of European, African, and Asian populations (3q26.1 and 16p.11.2) (EPICURE Consortium et al., 2012a,b; International League Against Epilepsy Consortium on Complex Epilepsies (ILAE Consortium on Complex Epilepsies), 2014; International League Against Epilepsy Consortium on Complex Epilepsies (ILAE Consortium on Complex Epilepsies)., 2018; Wang et al., 2019). Therefore, we may suggest that polygenic risk scores calculated in European populations at these specific loci could indeed be transferable to admixed Brazilian individuals.

However, although all these 29 candidate regions passed the Bonferroni correction based on the 48 candidate SNPs (*p-*value = 0.001), we understand that this *p*-value threshold is not stringent. Thus, the lack of association signal cannot be discarded for the 20 remaining candidate regions. Thus, one may still speculate that the lack of reproducibility could be due to the absence of statistical power, population stratification, or the differences in the genomic structure of the admixed sample compared with the previously studied populations.

Although we have identified flanking SNPs in the neighborhood of the candidate regions, which presented 80% of statistical power to detect increased risk or protection allele effect, we acknowledge the limited statistical power provided by the cohort analyzed, with 87 patients with GGE and 340 controls.

Despite the observed high heterogeneity within groups (σ^2^ = 99.61%) and low heterogeneity between patients and controls (σ^2^ = 0.39%), the statistics based on AMOVA results revealed evidence of population stratification between patients with GGE and the BIPMed controls. Thus, we corrected possible spurious association results by taking the first five principal components into account in the logistic regression model (Marchini et al., 2004; Price et al., 2010).

Indeed, the two-proportion Z-test showed that 16 SNPs presented different allele frequencies when comparing admixed Brazilian and European samples, further substantiating the hypothesis of lack of genetic association due to genetic differences when comparing the admixed Brazilians and Europeans.

It is important to note that 31 SNPs were not found in the SNP-array dataset, and we decided to impute them from all populations available in the 1KGP dataset (The 1000 Genomes Project Consortium et al., 2015). Previous studies have demonstrated that imputation accuracy for populations with a high proportion of European ancestry is higher than for populations with African or Native American ancestry (Martin et al., 2017). In addition, the EPIGEN-Brazil Initiative has also imputed admixed Brazilian samples from the 1KGP dataset with high confidence variants (Magalhães et al., 2018). However, the imputation by the TOPMED Consortium has demonstrated improved quality of variant imputation for admixed African and Hispanic/Latin populations compared with the 1KGP dataset (Kowalski et al., 2019). Thus, we also used this approach for comparison. We observed a perfect match between the SNPs genotyped in the SNP-array and their imputed correspondents for the BEAGLE imputation using the 1KGP as reference. By contrast, there was only 45.7% correspondence between the SNPs genotyped and the imputed SNPs using TOPMED. Thus, we can argue that Hispanic/Latin samples included in the TOPMED reference panel (Kowalski et al., 2019) may not represent the genomic structure of admixed Brazilians (Adhikari et al., 2016). This is an important finding and indicates that although allele frequencies of admixed Brazilian populations are different from other populations reported in public databases (Adhikari et al., 2016; Magalhães et al., 2018; Rocha et al., 2020), there is a remarkable accuracy in the SNP imputation for admixed Brazilian individuals based on populations from the 1KGP database, as demonstrated by our results and elsewhere (Magalhães et al., 2018).

In conclusion, we replicated association signals on eight candidate regions previously found in European populations, indicating the possibility of transferability of polygenic risk scores from European studies to admixed Brazilian populations in these specific candidate regions. In addition, we show evidence that differences in the genetic architecture of the population may hinder the replication of association results in admixed Brazilians for the remaining candidate regions, thus supporting the hypothesis of population differences influencing the association results in the present study. Also, we documented the effect of different methods/databases used for genotype imputation in admixed Brazilians. These results could be relevant to improving stratification risk estimation and future precision health applications in admixed Brazilian patients with GGEs and other complex disorders.

## Data Availability Statement

The datasets presented in this study can be found in online repositories. The names of the repository/repositories and accession number(s) can be found below: https://www.ebi.ac.uk/eva/, PRJEB39251; https://www.ebi.ac.uk/ena, PRJEB45235.

## Ethics Statement

The studies involving human participants were reviewed and approved by Comitê de Ética em Pesquisa da Universidade Estadual de Campinas. The patients/participants provided their written informed consent to participate in this study.

## Author Contributions

FK performed data processing, statistical analysis, and imputation. TA and PA performed data acquisition and SNP array genotyping. MA, CY, and FC performed the clinical analysis of GGE patients. FC and IL-C served as the principal investigators. RS conceptualized the work, created the study design, and served as a principal investigator. All authors reviewed and approved the final version of the manuscript.

## Conflict of Interest

The authors declare that the research was conducted in the absence of any commercial or financial relationships that could be construed as a potential conflict of interest.
